# Qishen Yiqi Drop Pill, a novel compound Chinese traditional medicine protects against high glucose‐induced injury in cardiomyocytes

**DOI:** 10.1111/jcmm.14527

**Published:** 2019-07-06

**Authors:** Shouyan Zhang, Hao Wang, Lixia Li, Xuewei Chang, Huifang Ma, Mingming Zhang, Xiaochun Qing, Lijun Zhang, Zhuo Zhang

**Affiliations:** ^1^ Department of Cardiology Luoyang Central Hospital Affiliated to Zhengzhou University, Luoyang Institute of Cardio‐cerebrovascular Diseases, Luoyang Key Laboratory of Cardiac‐cerebro Tissue Injury and Repair Luoyang China

**Keywords:** diabetes cardiomyopathy, high glucose injury, PI3K/Akt, Qishen Yiqi Drop Pill, traditional Chinese medicine

## Abstract

**Objective:**

Qishen Yiqi Drop Pill (QSYQ) has been recognized as a potential protective agent for various cardiovascular diseases. However, the effect of QSYQ in cardiac complications associated with diabetes is not clear currently. In this study, we investigate whether QSYQ could exert cardiac protective effects against high glucose‐induced injuries in cardiac H9c2 cells.

**Methods:**

H9c2 cells were exposed to 24 hours of high glucose in presence or absence of QSYQ and LY294002. Cell cytotoxicity, apoptosis, reactive oxygen species (ROS) generation, mitochondrial membrane potential and mitochondrial permeability transition pore (mPTP) opening were determined. Levels of bax, bcl‐2, p53, cleaved caspase‐3, PI3K and Akt were evaluated by Western blot.

**Results:**

Our data indicated that QSYQ significantly increased the cell viability and decreased cytotoxicity. By analysing the apoptotic rate as well as the expression levels of cytoapoptosis‐related factors including cleaved caspase‐3, bax, bcl‐2, and p53, we found that QSYQ could remarkably suppress apoptosis of cardiomyoblasts caused by high glucose. In addition, it also showed that QSYQ reduced the generation of ROS. We further found that QSYQ treatment could inhibit the loss of mitochondrial membrane potential and mPTP opening. Moreover, Western blot analysis showed enhanced phosphorylation of PI3K/Akt. The specific inhibitor of PI3K, LY294002 not only inhibited QSYQ induced PI3K/Akt signalling pathway activation, but alleviated its protective effects.

**Conclusions:**

In summary, these findings demonstrated that QSYQ effectively protected H9c2 cells against the series injuries due to high glucose at least partially by activating the PI3K/Akt signalling pathway.

## 1 INTRODUCTION

Diabetes mellitus (DM) is a worldwide public health problem. According to the latest data released by the International Diabetes Federation (IDF), the global diabetes patients rose to 415 million in 2015, accounting for about 8.3% of the total population of the adult population.^1^, ^2^ From 2012 to 2015, the number of deaths per year because of diabetes and its complications was as high as 1.5‐5.0 million.^3^ Among the many complications of diabetes, cardiovascular disease (CVD) is still the leading cause of death in diabetes mellitus patients.^4^ The Framingham heart study showed that the incidences of heart failure (HF) in men and women with diabetes were 2.4 and 5 times as high as that of healthy people, respectively.^5^ Moreover, the mortality of patients with heart failure complicated by diabetes increased significantly.^6^ In addition, it suggests that diabetes is a risk factor for cardiovascular diseases such as coronary heart disease and hypertension, and the metabolic state of its own disorder has direct damage to the myocardium. In 1972, Rubler found in four autopsy cases of diabetic patients that these patients had heart failure without obvious coronary artery or heart valve disease, congenital heart disease, hypertension or alcohol abuse, and he named this kind of heart disease as diabetes cardiomyopathy (DCM) for the first time.^7^ Later, epidemiological studies found that more than half of the diabetic patients were combined with diabetic cardiomyopathy, and the death/disability of the population accounted for about 50% of the total number of deaths and disability in all diabetes and its complications.^8^, ^9^ Accumulating evidence has suggested that hyperglycaemia, insulin resistance/hyperinsulinemia and abnormal fatty acid metabolism are the main pathophysiological mechanisms of DCM,^10^, ^11^, ^12^ among which hyperglycaemia has gradually been considered as the most important trigger.^13^ At present, the pathophysiology of DCM is still not fully understood and there is no effective therapy for it.

Qishen Yiqi Drop Pill (QSYQ) is a traditional Chinese medicine (TCM), which was approved by China Food and Drug Administration (CFDA) in 2003 for treating HF (Approval number of CFDA: Z20030139). Briefly, QSYQ is composed of four extracts of herb plants, namely Salvia Miltiorrhiza Bunge (“danshen” in Chinese), Panax notoginseng (“Sanqi” in Chinese), Astragalus membranaceus (Fisch.) Bunge (“huangqi” in Chinese) and Dalbergia odorifera T. Chen (“Jiangxiang” in Chinese). Previous studies about QSYQ mainly focused on its role in ischaemic cardiomyopathy, which showed that it could effectively attenuate myocardial fibrosis, prevent cardiac remodelling and promote angiogenesis.^14^, ^15^ Recently, one of its main components, Radix Astragalus membranaceus (Huangqi), has been found to protect cardiomyocytes against apoptosis induced by high glucose.^16^ However, it is not clear whether QSYQ can exert such protective effects. A number of studies have established the cellular model of DCM by using H9c2 cell line, which is generated from embryonic rat heart, to simulate DCM because of its near identical pathological responses compared with that of primary cardiomyocytes.^17^, ^18^, ^19^ Therefore, the aim of the current study is to verify whether QSYQ can protect cardiomyocytes against high glucose‐induced injuries and to explore the underlying mechanisms.

## 2 METHODS AND MATERIALS

### 2.1 Reagents and antibodies

Qishen Yiqi Drop Pill (lot number: 110708) was obtained from Tasly Pharmaceutical Co., Ltd. with 0.5 g per pouch. Dulbecco's modified Eagle's medium (DMEM), foetal bovine serum (FBS) and sterile 1 × PBS were purchased from Gibco (Carlsbad). Primary antibodies including cleaved caspase‐3 polyclonal rabbit antibody, bcl‐2 polyclonal rabbit antibody, bax polyclonal rabbit antibody, p53 monoclonal mouse antibody and β‐actin monoclonal mouse antibody, as well as LY294002, the specific inhibitor of PI3K, were obtained from Abcam. DCFDA Cellular ROS Assay Kit was also purchased from Abcam. Horseradish peroxidase‐conjugated secondary antibodies were obtained from Invitrogen. ECL chemiluminescence detection kit was obtained from Amersham Pharmacia Biotech. RIPA lysis buffer, BCA protein assay kit, TUNEL kit and the in vitro toxicology assay kit based on 3‐[4,5‐dimethylthiazol‐2‐yl]‐2,5‐diphenyltetrazolium bromide (MTT) were purchased from Sigma‐Aldrich. Polyvinylidene difluoride (PVDF) membranes were from Bio‐Rad. The MitoCapture mitochondrial apoptosis detection kit was obtained from BioVision. The CytoTox 96® non‐radioactive cytotoxicity assay kit was obtained from Promega. The goat anti‐rabbit Alexa Fluor 555 secondary antibody and 4,6‐Diamidino‐2‐phenylindole (DAPI) were obtained from Thermo Fisher Scientific.

### 2.2 Preparation of QSYQ solution

To prepare QSYQ stock solution (500 mg/mL), 0.5 g QSYQ pills (one pouch) were dissolved into 1.0 mL sterile 1 × PBS. Bullet blender (Next Advance, Inc) was used to ensure the pills be dissolved completely. Then, the solution was filtered through a Millex‐GP filter (filter unit, 0.22 µm), to remove bacteria and possible undissolved precipitations. When used, the QSYQ stock solution was diluted into culture medium to make working solution with different final concentration, including 5, 4, 3, 2, 1 mg/mL, 500, 250, 100 and 10 μg/mL.

### 2.3 Cell culture and treatments

The H9c2 cells, a rat cardiac myoblast cell line, were purchased from the Procell Life Science & Technology Co., Ltd. Cells were cultured in Dulbecco's Modified Eagle Medium (DMEM) (Gibco, Carlsbad) supplemented with 10% foetal bovine serum (FBS) at 37°C in an incubator with a humidified atmosphere of 5% CO_2_, and the medium was replaced every three days. Cells in the logarithmic growth phase were used for experiment. Cells incubated with routine low‐glucose culture medium (containing 5.5 mmol/L glucose) were used as control group. Cells were incubated with 1% FBS DMEM (containing 55 mmol/L glucose) for 24 hours as the high glucose damage model. In QSYQ treatment group, sterile QSYQ solution was added into culture medium to make different final concentration. In QSYQ + LY294002 group, both 10 μmol/L LY294002 and QSYQ were added into culture medium. After 24 hours incubation, cells were harvested for the examination of cell viability, cytotoxicity, apoptosis, ROS generation and mitochondrial function. Signalling pathways were analysed using Western blot analysis.

### 2.4 Determination of cell death

Cytotoxicity was detected via measuring lactate dehydrogenase (LDH) release in the culture medium by using the CytoTox 96® non‐radioactive cytotoxicity assay kit (Promega), which is described in the previous section.^20^ Briefly, lysis solution, supplied by the kit, was added to particular wells to generate the maximum LDH release control 45 minutes ahead, and then supernatants were taken (50 μL/well) after the intervention described above. After this, corresponding reagents were added according to manufacturer’s instructions. The plate was placed at room temperature for 30 minutes before stop solution was added. The OD value of each well was measured using a multimode plate reader (PerkinElmer Victor x3) at 490 nm. The mean optical density (OD) of three wells in the indicated groups was used to calculate the percentage of cytotoxicity according to the following formula: Per cent cytotoxicity = 100 × (Experimental‐Culture Medium Background)/(Maximum LDH Release‐Culture Medium Background). The experiment was repeated five times.

### 2.5 Measurement of cell viability

Cell viability was assessed using the in vitro toxicology assay kit (Sigma‐Aldrich) based on MTT and conducted according to the instructions.^20^, ^21^ At the end of the experiment, the medium was removed, and the cells were washed with 1 × PBS. Then, corresponding reagents (containing MTT) were added to each well. Cells were subsequently incubated for another 4 hours at 37°C. The medium was then abandoned, and 150 μL dimethyl sulfoxide (DMSO) was added to each well which was then shaken for 10 minutes at room temperature to completely dissolve the blue‐purple precipitate from the MTT. The OD value of each well was measured using a multimode plate reader (PerkinElmer Victor x3) at 570 nm. The values are expressed as percentages of low‐glucose group (LG) control values. The experiment was repeated five times.

### 2.6 Cell apoptosis assessment by immunochemical staining and TUNEL assay

Cell apoptosis was determined by both active caspase‐3 immunostaining as well as terminal deoxynucleotidyl transferase (TdT) nick‐end labelling (TUNEL). Immunostaining was performed as described previously.^20^, ^21^ At the end of treatment, cells were washed in 1 × PBS, fixed via immersion in 4% paraformaldehyde for 15 minutes and permeabilized with 0.1% Triton X‐100 for 10 minutes at room temperature. Cells were then washed three times with 1 × PBS, blocked with 1% BSA in 1 × PBS for 1 hour at room temperature and incubated with polyclonal anti‐active caspase‐3 antibody for 2 hours at a dilution of 1:100 at room temperature. Following three washes with 1 × PBS, cells were incubated with goat anti‐rabbit Alexa Fluor 555 secondary antibody (Life Technologies) in 1 × PBS for 1 hour at room temperature. Cells were then counterstained with DAPI to visualize the nuclei. Images were captured using a fluorescence microscopy (Leica DMI4000B). The percentage of apoptotic positive cells was determined in five randomly chosen fields and was normalized with the total number of stained nuclei. Terminal deoxynucleotidyl transferase (TdT) nick‐end labelling Cell apoptosis was analysed using TUNEL kit from Sigma‐Aldrich. Briefly, cells were washed with cold 1 × PBS for three times, fixed with 4% paraformaldehyde for 15 minutes and permeabilized by incubation in 1 × PBS containing 0.1% Triton X‐100 for 10 minutes at room temperature. Cells were then washed three times with 1 × PBS and incubated with the labelling solution (containing fluorescein‐dUTP) for 1 hour at 37°C in dark according to the manufacturer's instruction. Finally, nucleus was stained with DAPI. Apoptotic cells were observed using a fluorescence microscope (Leica DMI4000B).

### 2.7 Western blot

In brief, cells were lysed in cold RIPA buffer after experiments, and protein was separated by centrifugation. BCA Protein Assay Kit (Sigma‐Aldrich) was used for the determination of protein concentration. Loading buffer was added to the cytosolic extracts and then heated on at 100°C for 5 minutes; the same amounts of supernatant from each sample were subjected to 10% or 12% sodium dodecyl sulphate‐polyacrylamide gel electrophoresis (SDS‐PAGE), and then the total number of proteins was transferred onto PVDF membranes. The membranes were blocked overnight in 5% fat‐free milk at 4°C and incubated with their respective antibodies, including active caspase‐3 polyclonal, bcl‐2 polyclonal, bax polyclonal, p53 monoclonal, PI3K polyclonal, phosphor‐PI3K (p‐PI3K), Akt polyclonal, phosphor‐Akt (p‐Akt) polyclonal and β‐actin monoclonal antibodies at the recommended diluted concentration, then visualized by anti‐rabbit or anti‐mouse horseradish peroxidase‐conjugated secondary antibody (1:2,000) and finally developed with ECL chemiluminescence detection reagent (Amersham Pharmacia Biotech). In order to quantify the protein expression levels, the X‐ray films were scanned and analysed using ImageJ software (Wayne Rasband, NIH). The experiment was carried out 4‐5 times.

### 2.8 Determination of mitochondrial membrane potential

The mitochondrial membrane potential (MMP) was indirectly determined using a MitoCapture kit from BioVision. Firstly, cells from different treatment groups were washed with 1 × PBS and then incubated with MitoCapture^™^ Reagent, which was diluted in advance using incubation buffer supplied by the kit at a concentration of 1:1000, for 20 minutes at 37°C protected from light. Then, remove the medium, add new incubation buffer and observe under a fluorescence microscope (Leica DMI4000B) immediately. The red fluorescent signals were excited at 530 nm and detected at 630 nm, and the green fluorescence was excited at 488 nm and detected at 530 nm, respectively.

### 2.9 Mitochondrial permeability transition pore

The MitoProbe™ transition pore assay kit from Thermo Fisher Scientific was used to evaluate the opening of mitochondrial permeability transition pore (mPTP). According to manufacturer’s instructions, cells were washed three times with Hanks' balanced salt solution‐10 mmol/L HEPES (pH 7.2) after all treatments and then incubated with 500 nmol/L calcein‐AM for 20 minutes at 37°C protected from light. After incubation, CoCl_2_ was added to quench the cytoplasmic signal so that only the fluorescence mitochondria were captured, as in healthy cells CoCl_2_ could not enter mitochondria. The cells were observed under a fluorescence microscope immediately. Integrated optical density was analysed by ImageJ software (Wayne Rasband, NIH) and change in fluorescence intensity is an index of mPTP opening. The experiment was carried out 4‐5 times. Results are expressed as percentages of LG group.

### 2.10 Measurement of intracellular ROS generation

DCFDA Cellular ROS Assay Kit was employed to detect the production of intracellular reactive oxygen species (ROS). Briefly, cells were digested and harvested after treatment, and then stained with 20 μmol/L DCFDA for 30 minutes at 37°C. After staining, cells were gently pipetted up and down to ensure single‐cell suspension. Finally, cells were analysed via a flow cytometer (BD FACSCanto™ Flow Cytometer). DCF is excited by the 488 nm laser and detected at 535 nm. A minimum of 30 000 cells were analysed per experimental condition, and results were expressed as the fold change of the mean fluorescence intensity (MFI) over the LG control group.

### 2.11 Statistical analysis

All data are expressed as mean ± SEM. Differences among groups were detected by one‐way analysis of variance (ANOVA), followed by Bonferroni correction. A value of *P *< 0.05 was considered statistically significant.

## 3 RESULTS

### 3.1 QSYQ attenuated cytotoxicity of cells exposed to high glucose

**Figure 1:**
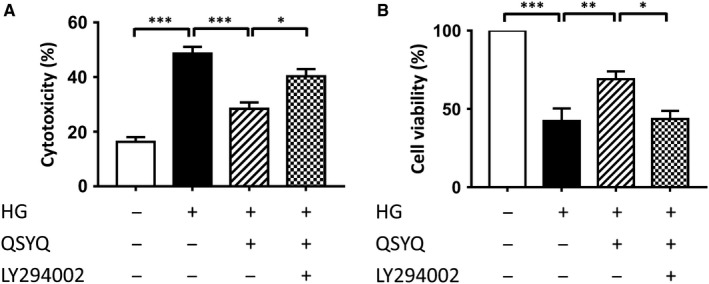
Effects of QSYQ on cell cytotoxicity and viability. A, QSYQ attenuated cytotoxicity after high glucose injury. B, QSYQ increased cell survival in cardiomyoblasts exposed to high glucose injury. Values represent means ± SEM (n = 4‐5/group). *P < 0.05, **P < 0.01, ***P < 0.001

To examine the effects of QSYQ on HG‐induced cytotoxicity, cell death by necrosis was examined by measuring the release of cytosolic LDH enzyme. We carried out these measurements after subjecting cells to 24 hours of high glucose injury. As shown in Figure 1A, LDH release increased from 16.26 ± 1.75% to 48.57 ± 2.53% in response to high glucose (*P* < 0.001). QSYQ treatment remarkably mitigated high glucose‐induced cell deaths, as evident by the great reduction of LDH leakage to 28.39 ± 2.35% (*P* < 0.001). However, this decrease was partially blocked by LY294002, in which group approximately 40.29 ± 2.65% cells died (*P* = 0.015).

### 3.2 QSYQ increased cell survival and rescued high glucose‐induced injury in cardiomyoblasts

As compared with the low‐glucose condition, cardiomyocytes exposed to high glucose demonstrated cellular injury, as evident by the decrease in MTT. QSYQ treatment significantly increased the magnitude of MTT in response to high glucose, which is shown in Figure 1B. After HG, H9c2 cell viability was decreased to 42.33 ± 7.92% (*P* < 0.001), which was prevented with treatment of QSYQ at 1 mg/mL with cell viability of 69.04 ± 4.93% (*P* = 0.006). However, this protective effects were neutralized by the interference of LY294002, the PI3K specific inhibitor (*P* = 0.006). Besides, higher or lower dose of QSYQ treatment (2 mg/mL, 500, 250, 100 and 10 μg/mL) did not elicit significant protective effects in this model (data not shown). Moreover, when QSYQ concentration exceeded 2 mg/mL, cells begun to shrink because of high permeating press.

### 3.3 QSYQ suppressed high glucose‐induced cell apoptosis

**Figure 2:**
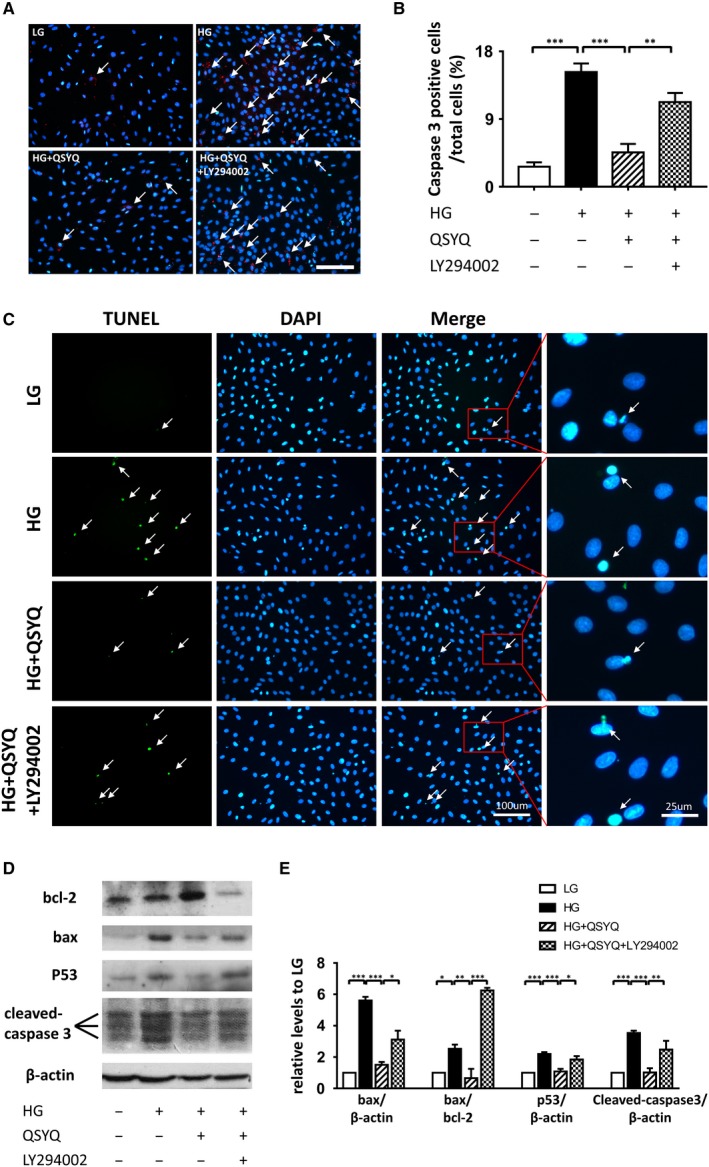
QSYQ attenuated cell apoptosis induced by high glucose injury. A, Representative images showing the apoptotic H9c2 cardiomyoblasts: active caspase‐3 positive nuclei in red (white arrows); nuclei were stained in blue (DAPI). B, Quantification of active caspase‐3 positive nuclei between groups. Values represent means ± SE (n = 5/group). **P < 0.01, ***P < 0.001. Scale bar: 100 μm. C, Representative micrographs of apoptotic cells with TUNEL staining. Apoptosis cells were identified by TUNEL staining (green) (white arrows), and total nuclei by DAPI staining (blue). QSYQ significantly improved cytoapoptosis induced by high glucose and LY294002 reversed this protective effect. D, Expression levels of bcl‐2, bax, p53, and cleaved caspase‐3 were detected by Western blot. QSYQ remarkably suppressed the increase of cytoapoptosis factors levels resulted from high glucose injury. E, Relative intensity of protein levels were quantified using densitometric analysis. Values represent means ± SEM (n = 4‐5/group). *P < 0.05, **P < 0.01, ***P < 0.001

The impact of QSYQ on apoptosis of H9C2 cells after high glucose treatment was assessed by both active caspase‐3 staining and TUNEL assay. As shown in Figure 2A,B, the apoptosis ratio of cells in the HG group was much higher than that of the LG group, which increased from 2.66 ± 0.59% in LG to 15.25 ± 1.14% in response to high glucose (*P* < 0.001). The application of QSYQ significantly decreased the number of active caspase‐3 positive cells (4.57 ± 1.12% in the HG + QSYQ group, *P* < 0.001). However, cell apoptosis rate increased to 11.26 ± 1.20% again after administration of LY294002 (*P* = 0.001). In addition, we also found some morphological changes such as rippled or creased nuclear, and apoptotic bodies in Figure 2A. Therefore, TUNEL assay was conducted to visualize the existence of fragmented DNA generated during cell apoptosis as well as increase the robustness of these microscopic experiments, which also showed that QSYQ could prevent cytoapoptosis caused by high glucose, and LY294002 neutralized it (Figure 2C). To verify further the effect of QSYQ on alleviating apoptosis induced by HG, we detected the expression of key apoptosis‐related proteins, including cleaved caspase‐3, bcl‐2, bax and p53. Compared with HG group, the levels of cleaved caspase‐3, p53 and bax, as well as the ratio of bax to bcl‐2, decreased obviously after QSYQ treatment. However, addition of LY294002 abolished the cardioprotective effect of QSYQ, as displayed in Figure 2D,E.

### 3.4 QSYQ protects against high glucose‐induced mitochondrial damage

**Figure 3:**
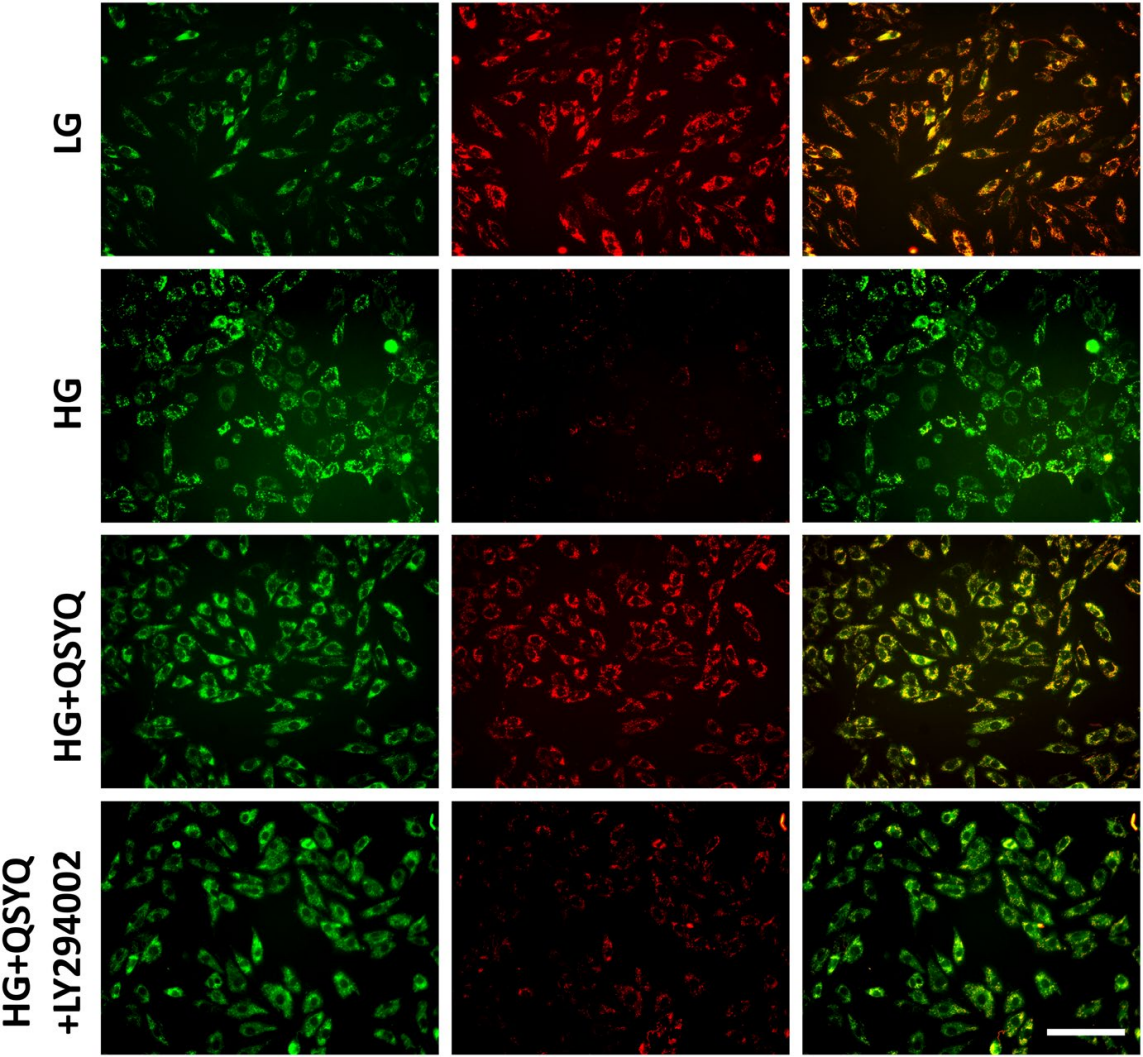
The effect of QSYQ on high glucose‐induced MMP reduction in H9c2 cells. Cardiomyoblast mitochondrial damage was assessed by examining mitochondrial membrane depolarization which had been described in detail in materials and methods. Mitochondrial apoptosis was more severe in the high glucose group. However, QSYQ treatment improved the high glucose‐led MMP loss significantly and this effect was partially blocked by LY294002. Scale bar: 100 μm

Modification of the mitochondrial membrane potential (MMP) is an early event in the induction of apoptosis. As shown in Figure 3, exposure of H9c2 cardiomyoblasts to high glucose caused a remarkable decrease in MMP, as evident by the significant loss of red fluorescent signals in HG group compared to the low‐glucose condition. Administration with QSYQ significantly reversed the high glucose‐induced disruption of MMP. But the effect of QSYQ was blocked by coadministration of LY294002.

### 3.5 QSYQ inhibited the mPTP opening

**Figure 4:**
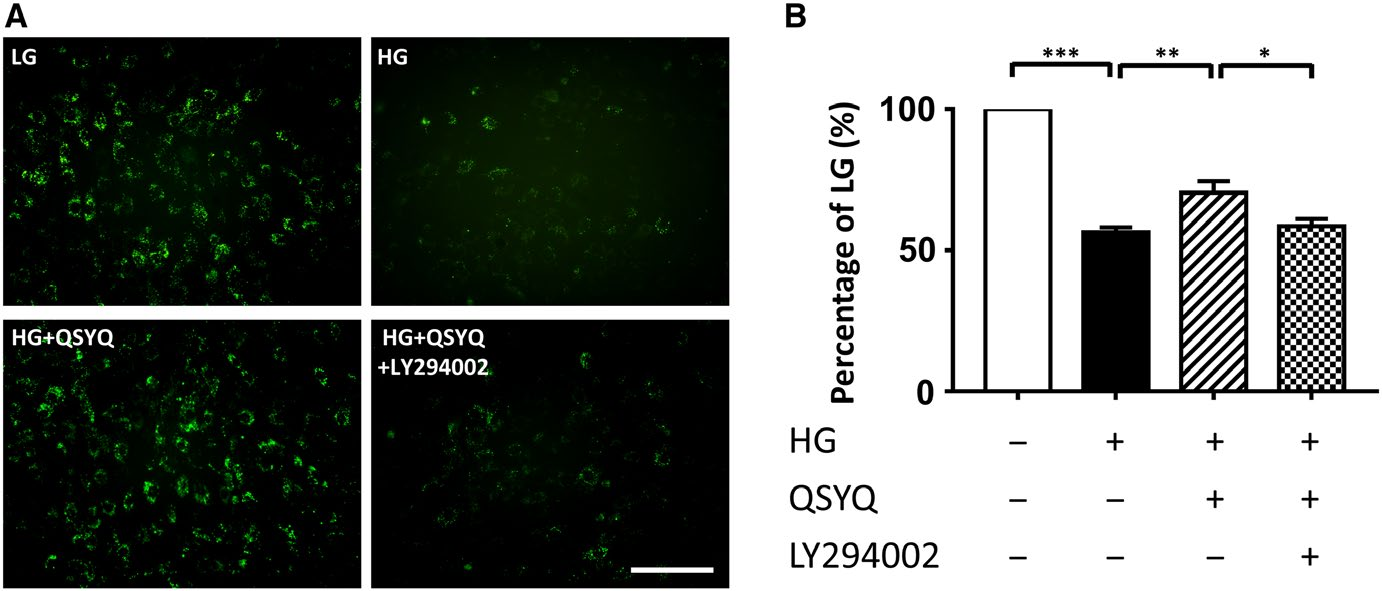
Effects of QSYQ on mitochondrial permeability transition pore (mPTP) opening in cardiomyoblasts exposed to high glucose. A, Representative images of mPTP staining. Scale bar: 100 μm. B, Quantitation analysis of mPTP in H9c2 cardiomyoblasts exposed to high glucose. Our analysis showed that QSYQ treatment rescued mPTP opening led by high glucose. The results represent 4‐5 independent experiments counting 150 ± 200 cells per condition. Values represent means ± SE (n = 4‐5/group). *P < 0.05, **P < 0.01, ***P < 0.001

The opening of mPTP in H9C2 cells was directly assessed using calcein‐AM assay (Figure 4A,B). After the sequence of high glucose injury, the green fluorescence of calcein was maintained in a significantly higher level in cells incubated with QSYQ than that absence of QSYQ treatment, indicating that QSYQ inhibited the opening of mPTP, which was also reversed by LY294002.

### 3.6 QSYQ reduced intracellular ROS generation

**Figure 5:**
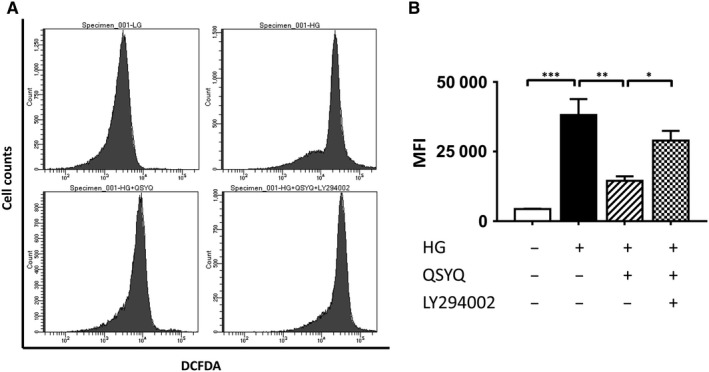
Effects of QSYQ treatment on ROS generation assessed by flow cytometry in H9c2. A, Representative images of flow cytometry. B, The quantitative flow cytometry results show that QSYQ notably inhibited ROS generation as evident by the reduction in MFI. Values represent means ± SE (n = 4‐5/group). *P < 0.05, **P < 0.01, ***P < 0.001

The effect of QSYQ on the intracellular ROS production induced by high glucose was shown in Figure 5A,B. Cells in HG group produced stronger fluorescence signals than that in LG group. Yet, incubation with QSYQ resulted a marked reduction in MFI, and this decrease was also reversed by the co‐administering of LY294002.

### 3.7 QSYQ promoted PI3K/AKT phosphorylation

**Figure 6:**
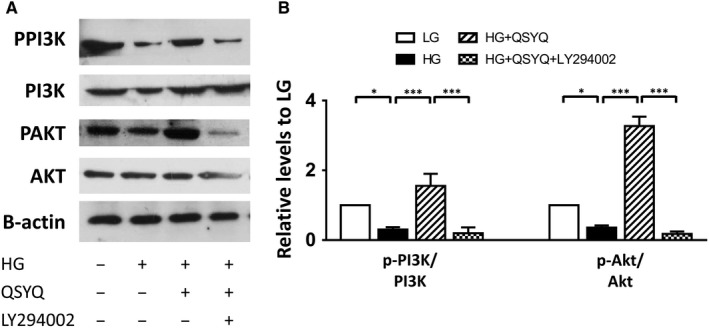
QSYQ treatment induced alterations in PI3K/Akt signaling pathway in myocardium. A, Western blot showing Akt, p‐Akt, PI3K and p‐PI3K expression levels. B, Relative intensity of protein levels were quantified using densitometric analysis. It show that phosphorylation of Akt and PI3K signals were significantly enhanced by QSYQ treatment. Values represent means ± SE (n = 4‐5/group). *P < 0.05, ***P < 0.001

Dysregulation of the PI3K/Akt pathway is implicated in a number of human diseases including cancer, diabetes, cardiovascular disease and neurological diseases. In our study, no significant change in the expression levels of PI3K and Akt has been observed in each group. But the phosphorylation levels of PI3K (p85αTyr607) and Akt (Thr308) notably increased by QSYQ treatment, which were slightly reduced in HG group (Figure 6). Akt is a major mediator of cell survival through direct inhibition of pro‐apoptotic proteins, so it is in accordance with the results we obtained above. All these data demonstrated that QSYQ protected H9c2 cells against high glucose injury at least partially via activation of PI3K/Akt pathway.

## 4 DISCUSSION

In the current study, we investigated the protective effect of QSYQ, a novel TCM, from myocardial HG injury using an in vitro model. Briefly, H9C2 rat cardiomyoblasts were exposed to HG, which physiologically mimicked DCM injury to myocardium. Our results strongly suggest the cardioprotective effect of QSYQ from HG injury, which was mainly manifested on the decreases of cytotoxicity, cell apoptosis, suppression of ROS generation and the notable increase of cell viability as well as the effective prevention of mitochondrial dysfunction. In the preliminary exploration of mechanisms, we found that QSYQ might generate the protective effects in H9c2 cells exposed to high glucose through activation of PI3K/Akt signalling pathway.

TCMs have been used in the treatment of cardiac diseases for thousands of years, and many drugs have been proven effective.^22^, ^23^, ^24^, ^25^ QSYQ is a patent prescription of TCM, which has been approved for clinical application. Current studies, including clinical trials, animal and cellular experiments, indicated that QSYQ has a definite cardiac protective effect in ischaemic heart diseases, which can reduce apoptosis, suppress oxidative stress, alleviate inflammation, promote angiogenesis, attenuate myocardial fibrosis and finally improve cardiac function.^15^, ^26^, ^27^, ^28^ Chen et al^29^ found that the impaired myocardial mitochondrial structure and decreased level of ATP (accompanied by reduction of ATP5D and increase in the expression of cytochrome C), myocardial fibre rupture, interstitial oedema and infiltrated leucocytes induced by ischaemia/reperfusion injury were all significantly ameliorated by pretreatment with QSYQ. There are many common pathophysiological processes between IR injury and HG‐induced injury. In our study, we also found that QSYQ can suppress MMP loss and inhibit mPTP opening, maintaining mitochondrial function. Besides these findings, we know the major active ingredients of QSYQ are ginsenosides Rg1 and Rb1 (from Sanqi), astragaloside (from Huanqi), and tanshinol (from Danshen).^30^, ^31^ Though no study directly explores the possible protective effect of QSYQ in DCM, there are many studies showed that its major active ingredients including ginsenosides Rb1, tanshinol and astragaloside can protect heart against diabetic cardiac complications.^32^, ^33^, ^34^ To the best of our knowledge, this study is the first demonstration that QSYQ protected the heart against HG injury.

In the exploration for mechanisms, we found that the cardiac protective effects of QSYQ could be partially reversed by LY294002, the specific PI3K inhibitor. It is well known that the activation of the PI3K/Akt signalling pathway can inhibit HG‐induced apoptosis.^35^ The energy substrate utilization shift from glucose to FFA oxidation has been known to contribute to the pathogenesis of DCM.^36^ PI3K/Akt pathway participated in physiological and pathological processes such as regulation of glycogen synthesis and disposal.^37^ In the present study, Akt and PI3K phosphorylation were observed notably decreased in the HG group as compared with the LG group, which is in accordance with the previous report.^38^ Furthermore, Akt exerts a particularly important impact on the regulation of glucose metabolism and supports cell survival by restraining apoptosis via activation or inactivation of a number of target proteins involved in the process of apoptotic cascades.^39^ In this study, cardiomyocytes treated with QSYQ showed a remarkable increase in expression of Akt and PI3K phosphorylation. These results provide evidence showing that PI3K/Akt signalling pathway might be involved in the inhabitation of cardiac HG injury induced by QSYQ.

Because the current study is a pilot study of our following animal experiments, it mainly focused on the phenomenon of the protective effects brought by QSYQ, while we only did preliminary exploration about the possible mechanisms, which is our limitation. We will make further improvement in our future animal studies. Due to the limitation of resources, we did not use advanced technologies such as single‐cell operation and RNA sequencing, which would be more precise and be able to detect vary of signalling pathways at the same time. This is another limitation for our study.

## 5 CONCLUSION

In conclusion, these results demonstrated that QSYQ protected cardiomyocytes against high glucose‐induced injury through the improvement of mitochondrial function and reduction of apoptosis, and this protective effect is at least partially mediated by PI3K/Akt‐related signalling pathway.

## CONFLICT OF INTEREST

The authors report no conflicts of interest.

## AUTHOR CONTRIBUTIONS

Conceptualization: SYZ, HW; Data curation: SYZ, HW, LXL, XCQ, LJZ; Formal analysis: XWC, MMZ, ZZ, XCQ; Funding acquisition: SYZ; Investigation: SYZ, HFM, HW, LXL, XWC, MMZ, XCQ, LJZ, ZZ; Methodology: HW, XWC, LJZ; Project administration: SYZ, HFM; Resources: SYZ; Supervision: SYZ; Validation: SYZ, HFM; Visualization: SYZ; Writing ± original draft: HW; Writing ± review & editing: SYZ, HFM.

## Data Availability

The data that support the findings of this study are available on request from the corresponding author.
